# Wilt disease reshapes rhizosphere microbiota in small yellow ginger soils

**DOI:** 10.3389/fmicb.2025.1670956

**Published:** 2025-10-21

**Authors:** Qing Sun, Sitian Fei, Sihao Huang, Runtao Tan, Haibo Chen, Songquan Song, Bin Wang

**Affiliations:** ^1^Nanling Research Institute for Modern Seed Industry, Xiangnan University, Chenzhou, China; ^2^School of Chemistry and Environmental Science, Xiangnan University, Chenzhou, China; ^3^R&D Centre of Rucheng Small Yellow Ginger Breeding Technology, Xiangnan University, Chenzhou, China; ^4^School of Basic Medical Sciences, Xiangnan University, Chenzhou, China; ^5^Institute of Botany, Chinese Academy of Sciences, Beijing, China

**Keywords:** ginger, wilt disease, *Ralstonia*, soil microbiota, microbial diversity

## Abstract

**Introduction:**

Continuous cropping obstacles, particularly the prevalent ginger wilt disease (bacterial wilt), severely constrain the sustainable development of the small yellow ginger (*Zingiber officinale* Roscoe) industry in Rucheng County, China. However, the primary pathogen responsible for this disease in the local cultivar and the associated microbiome shifts within the rhizosphere remain unidentified. This study aimed to elucidate the distinctive rhizosphere microbial community changes induced by ginger wilt disease, identify the potential key pathogen responsible for this disease in Rucheng, and provide a scientific basis for overcoming continuous cropping obstacles in small yellow ginger cultivation.

**Methods:**

Soil samples were collected from an uncultivated plot and from the rhizosphere of healthy and wilted small yellow ginger plants. Microbial community structure and composition were analyzed using 16S rRNA gene high-throughput sequencing. Linear Discriminant Analysis (LDA) effect size (LEfSe) was employed to identify differential biomarkers, and functional prediction was performed using BugBase.

**Results:**

Ginger wilt disease significantly altered the rhizosphere soil bacterial community structure and composition: the relative abundance of Proteobacteria increased significantly, primarily due to the enrichment of the genus *Ralstonia*; conversely, the relative abundance of Acidobacteriota, Firmicutes, and Chloroflexi significantly decreased. Ginger wilt disease also significantly reduced the diversity of the rhizosphere soil bacterial community. LEfSe further confirmed *Ralstonia* as a diagnostic biomarker for ginger wilt disease. BugBase phenotypic prediction indicated that the microbiota enriched in the diseased ginger rhizosphere exhibited higher capabilities for oxidative stress resistance, pathogenic potential, and mobile element content, attributed to a functional consortium of multiple genera, such as *Ralstonia* as the dominant contributor alongside major contributors such as *Rhodanobacter* and *Dokdonella*.

**Discussion:**

Our findings highlight that the enrichment of *Ralstonia* is strongly associated with ginger wilt in Rucheng County and concomitant with profound changes in the rhizospheric microbiota of wilted ginger, involving alterations in both community structure and functional potential.

## 1 Introduction

Ginger (*Zingiber officinale* Roscoe) is one of the most important economic crops in the world, which is widely used as food, medicine, and cosmetic additives (Benzie and Wachtel-Galor, 2011; Zhang et al., 2021). In China, ginger cultivation spans multiple regions, with Shandong, Yunnan, and Hunan provinces being particularly known for their diverse and distinctive local varieties. Nevertheless, ginger production is severely affected by a wilt disease, which is usually caused by the pathogen *Ralstonia solanacearum* (Paret et al., 2008; Prameela and Suseela Bhai, 2020; Dang et al., 2023). This devastating pathogen directly reduces yields by 40–50% in endemic areas, with complete crop failure occurring in severe epidemics (Guji et al., 2019; Aysanew and Alemayehu, 2022). Additional pathogens implicated in wilt disease include *Achromobacter xylosoxidans* (Hua et al., 2024), *Enterobacter cloacae* (Nishijima et al., 2004; Wang et al., 2008; Liu et al., 2021; Zhao et al., 2022), and *Ceratocystis fimbriata* (Zhang et al., 2020). The persistence of these pathogens in soil often forces farmers to abandon infected fields in search of new lands for cultivation, creating a critical situation. In Rucheng County, a leading production area for high-quality small yellow ginger in southern Hunan Province, wilt disease poses a serious threat to the local ginger industry, yet the primary pathogen remains unidentified.

Soil microorganisms are the linchpin of soil fertility, affecting not only soil health but also crop disease resistance and agricultural product quality. Rhizosphere microbes play a pivotal role in nutrient cycling and disease suppression, highlighting the significance of microbial diversity and abundance as critical factors for successful cultivation (Bai et al., 2022; Liu et al., 2023; Saud et al., 2023). Continuous monoculture can disrupt microbial community balance, often reducing microbial diversity and promoting disease outbreaks (Li et al., 2021; Haq et al., 2023). While crop rotation is a traditional strategy to mitigate the continuous cropping obstacles (Haq et al., 2023; Liu et al., 2024), the ginger–rice rotation practiced in Rucheng has proven ineffective against wilt disease.

Despite its agricultural significance, ginger’s continuous cropping obstacles and associated shifts in soil microbiota remain poorly understood. A preliminary study in Chongqing, China, reported microbial differences between soils of healthy and disease-affected ginger plants (Liu et al., 2017). Similarly, a study in Taiwan observed microbial community shifts after pathogen infection; however, that study focused primarily on the influence of biocontrol agents and fungicides on the microbiota rather than a direct comparison between healthy and diseased soils (Wang et al., 2022a). Furthermore, an empirical study demonstrated that *Ralstonia solanacearum* infection can enhance ginger resistance by inducing antibacterial root exudates and enriching beneficial bacteria (Dang et al., 2023). Nevertheless, a detailed comparative analysis of the microbiota in wilt-affected and non-wilt-affected soils is still lacking, and the influence of ginger cultivation and disease progression on the soil microbial composition is not well defined. Consequently, a thorough assessment of soil microbial communities during ginger cultivation and disease incidence is imperative.

This study employs high-throughput 16S rRNA gene sequencing to analyze the diversity of soil bacterial communities in Rucheng County. The goal is to elucidate the influence of ginger cultivation and wilt disease on soil microbial communities, identify the potential pathogen responsible for ginger wilt, and offer insights into the mechanisms that could alleviate the continuous cropping constraints faced by ginger farmers.

## 2 Materials and methods

### 2.1 Site description

The study was conducted at a site in Rucheng County, Chenzhou City, Hunan Province (25°30′56″N, 113°36′38″E). The study area falls within a subtropical monsoon humid climate zone, characterized by an average temperature of 16.6 °C, an average annual sunshine duration of 1,731 h, and an annual precipitation of 1545.7 mm. The ginger variety under investigation is the renowned “Rucheng small yellow ginger,” a local cultivar celebrated for its distinctive qualities and adaptability to the region’s unique environmental conditions.

### 2.2 Soil sample collection

To minimize confounding effects from soil heterogeneity, all ginger rhizosphere soil samples were collected from a single, continuous “small yellow ginger” cultivation field. Soil samples were divided into three groups: soils from three different locations within a continuous uncultivated area adjacent to the field (50–100 cm from the field edge), serving as the Control group (Group C); rhizosphere soil samples from healthy ginger plants, representing the Non-wilt-affected group (Group N); and rhizosphere soil samples from wilted ginger plants, defined as the Sick group (Group S). Each group consisted of three biological replicates. The three replicates for Groups N and S were collected from three independent healthy or wilted plants spaced more than 5 m apart within the same field. This design ensures that observed microbiome differences between N and S groups are primarily driven by plant health status rather than field-scale environmental variation. Sampling was conducted on 28 July 2022.

The rhizosphere soil was collected by carefully excavating around small yellow ginger plants to a depth of 0–20 cm, preserving the complete root systems. Non-adhering soil was gently removed, while soil within 0–0.5 cm of the roots was taken as the rhizosphere sample. After collection, the soil was homogenized, placed in sterile bags, and stored at −80 °C for future analysis (Zhang, 2024).

### 2.3 DNA extraction and sequencing

Total DNA was extracted from 0.5 g of soil using the TGuide S96 Magnetic Soil/Stool DNA Kit (Tiangen Biotech, Beijing, China) following the manufacturer’s instructions. The DNA concentration was quantified with the Qubit dsDNA HS Assay Kit (Life Technologies, Gaithersburg, MD, United States) and Qubit 4.0 Fluorometer (Invitrogen, Thermo Fisher Scientific, Oregon, United States). The V3–V4 region of the bacterial 16S rRNA gene was amplified using universal primer pairs 338F/806R (338F: 5′-ACTCCTACGGGAGGCAGCA-3′; 806R: 5′-GGACTACHVGGGTWTCTAAT-3′) to characterize the composition and structure of the bacterial community (Wang et al., 2022b; Yang et al., 2024). The primers were tailed with sample-specific Illumina index sequences to allow for deep sequencing. The amplification reaction system (total volume, 10 μL) contained 5 μL of KOD FX Neo Buffer, 2 μL of 2 mM dNTPs, 0.3 μL of 10 μM forward primer, 0.3 μL of 10 μM reverse primer, 0.2 μL of KOD FX Neo, 5–50 ng of DNA template, and ddH_2_O to a final volume of 10 μL. The PCR cycle comprised initial denaturation at 95 °C for 5 min, followed by 25 cycles of denaturation at 95 °C for 30 s, annealing at 50 °C for 30 s, and extension at 72 °C for 40 s, with a final extension at 72 °C for 7 min. After amplification, the PCR products were purified using Agencourt AMPure XP Beads (Beckman Coulter, Indianapolis, IN, United States) and quantified by the Qubit dsDNA HS Assay Kit and Qubit 4.0 Fluorometer. After quantification, all amplicons were pooled in equal amounts. For library construction, sequencing was performed on an Illumina NovaSeq 6,000 platform (Illumina, San Diego, CA, United States).

### 2.4 Sequence data analyses

The raw paired-end (PE) sequencing data were in FASTQ format. First, raw data were filtered based on nucleotide quality using Trimmomatic (version 0.33) (Bolger et al., 2014) with the following parameters: ILLUMINACLIP:TruSeq3-PE.fa:2:30:10 LEADING:3 TRAILING:3 SLIDINGWINDOW:4:20 MINLEN:50. Subsequently, primer sequences were identified and removed using Cutadapt (version 1.9.1) (Martin, 2011), with parameters allowing up to 20% mismatches and a minimum overlap of 15 bp (-e 0.2 -O 15). The clean reads were then processed using the DADA2 pipeline (version 1.26.0) (Callahan et al., 2016) in R for sample inference, which included quality filtering, denoising, merging of paired-end reads, and chimera removal. Specifically, the filterAndTrim function was used to filter reads with the maxEE parameter set to 2, and other parameters were set to their default values. The error model was built from the data using the learnErrors function. Denoising was performed with the dada function, followed by merging of paired-end reads using the mergePairs function (with parameters: minOverlap = 18, maxMismatch = 0.2 × minOverlap). Chimeric sequences were identified and removed with the removeBimeraDenovo function using the “consensus” method. Subsequently, the Amplicon Sequence Variants (ASVs) table was filtered prior to taxonomic assignment to remove any ASVs with a total count less than two across all samples. Taxonomy annotation of the ASVs was performed using the “q2-feature-classifier” plugin in QIIME2 (Bolyen et al., 2019). A pre-trained Naive Bayes classifier based on the SILVA 138.1 database (Quast et al., 2013), specific to the 338F/806R primer pair targeting the V3-V4 region, was used with the “classify-sklearn” method under a confidence threshold of 70%.

### 2.5 Bioinformatic analysis

The bioinformatics analysis of this study was performed with the aid of the BMK Cloud (Biomarker Technologies Co., Ltd., Beijing, China). Prior to analysis, all samples were rarefied to an even sequencing depth (16S:32,087 sequences per sample) for alpha and beta diversity calculations and a clustered heatmap generation. All other analyses, such as abundance analysis, ternary plot analysis, LEfSe, and BugBase phenotypic prediction, were performed using the non-rarefied data.

Alpha diversity indices (Chao1, ACE, Shannon, Simpson) were calculated using QIIME2 to estimate microbial richness and evenness within each sample (Hagerty et al., 2020). Beta diversity was assessed using Bray–Curtis dissimilarity to measure the similarity among microbial communities across different samples, as implemented in QIIME (Caporaso et al., 2010). Further exploration of beta diversity was achieved through a suite of methods, such as principal coordinate analysis (PCoA), visualization via heatmaps, and non-metric multidimensional scaling (NMDS).

To identify microbial biomarkers significantly associated with wilt-diseased ginger plants, the LEfSe approach was employed (Segata et al., 2011). LEfSe integrates linear discriminant analysis (LDA) with non-parametric Kruskal–Wallis and Wilcoxon rank-sum tests to discern differentially abundant features between groups (Segata et al., 2011; Li et al., 2023). This method was chosen for its ability to account for microbial taxonomic hierarchy and identify consistently enriched taxa across phylogenetic levels. Features with an LDA score > 4.0 were considered discriminative.

To gain functional insights into the potential ecological impacts of the observed microbial community shifts, the organism-level microbiota phenotypes were predicted using BugBase software (Ward et al., 2017). The predicted phenotypes included Gram-positive, Gram-negative, biofilm-forming, pathogenic potential, mobile element-containing, oxygen-utilizing (aerobic, anaerobic, and facultatively anaerobic), and oxidative stress-tolerant. BugBase was run using its default parameters, which employ a data-driven approach to automatically determine the minimum trait coverage threshold for each phenotype by selecting the threshold that maximizes variance across all samples in the dataset.

### 2.6 Statistical analysis

Taxon abundances (at the phylum and genus levels), bacterial community alpha-diversity, and BugBase phenotypic predictions were analyzed using one-way analysis of variance (ANOVA) with the Bonferroni test (Mukhtar et al., 2021). Given the marked variance heterogeneity in the *Ralstonia* abundance and Simpson’s index, the non-parametric Kruskal–Wallis (KW) test and Dunn’s *post hoc* test were used to evaluate intergroup differences. The statistical significance of group clustering in the beta diversity analysis was tested using permutational multivariate analysis of variance (PERMANOVA; Adonis test) with 999 permutations in QIIME2. A significance threshold of a *p* value of <0.05 was applied for all statistical tests.

## 3 Results

### 3.1 Amplicon sequencing and species (ASV) composition

The soil samples (*n* = 9) were subjected to 16S rRNA gene sequencing to characterize the bacterial communities. Specifically, the V3–V4 regions of the bacterial 16S rRNA genes were amplified and assessed via high-throughput sequencing technology. A total of 1,091,200 raw reads were generated from these nine samples. Following quality control and primer removal, 1,087,336 clean reads were obtained. Further refinement through denoising, merging, and chimera removal culminated in a dataset comprising 663,121 high-quality reads (Table 1). These sequences were further denoised and then clustered into ASVs, which were annotated with taxonomic designations, resulting in the identification of 15,496 distinct ASVs.

**Table 1:** Sequencing data processing results statistics.

Sample ID	Raw reads	Clean reads	Denoised reads	Merged reads	Non-chimeric reads
C1	165,704	165,162	156,193	125,725	98,040
C2	79,972	79,770	73,863	58,871	49,221
C3	68,421	68,255	63,326	50,778	43,728
N1	55,073	54,759	50,513	37,878	32,240
N2	154,693	154,143	145,188	114,768	92,646
N3	138,574	138,029	129,321	101,099	81,612
S1	80,190	79,870	74,641	57,066	44,653
S2	147,795	147,258	140,422	115,237	89,880
S3	200,778	200,090	191,552	164,588	131,101
Total	1,091,200	1,087,336	1,025,019	826,010	663,121

The rarefaction analysis illustrates a leveling off of the rarefaction curves upon reaching a certain sequencing depth for all samples (Supplementary Figure S1). This suggests that the sequencing depth was sufficient to capture the majority of the species present within the samples, indicating a high level of coverage and validating the quality of the sequencing data (Xu et al., 2019). Consequently, the sampling depth and sequencing data volume were adequate to provide a reliable representation of the bacterial populations within the soil samples collected from small yellow ginger cultivation sites.

### 3.2 Abundance analysis of soil bacterial species

**Figure 1:**
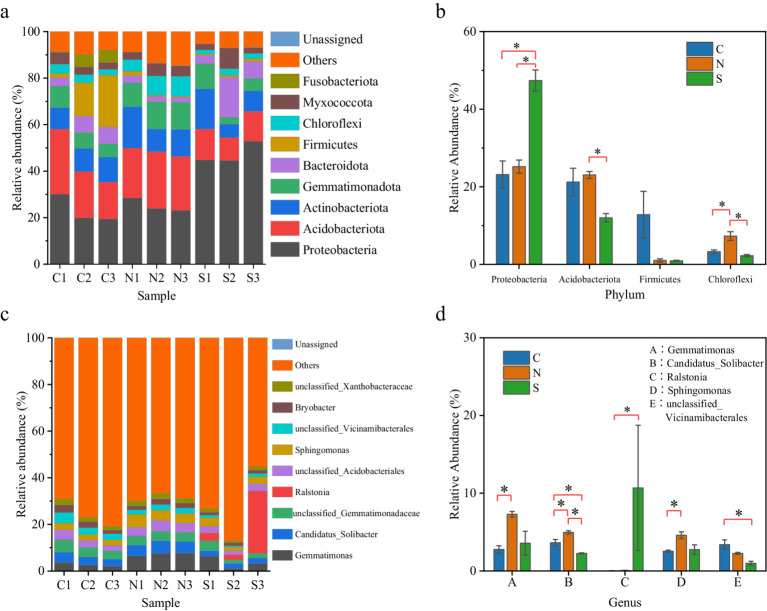
**(a)** The bacterial community composition at the phylum level. The horizontal coordinate refers to each group of ginger soil bacteria. The vertical coordinate refers to the relative abundance of each group. **(b)** The relative abundance comparison of the phyla Proteobacteria, Acidobacteriota, Firmicutes, and Chloroflexi among different samples. **(c)** Main community composition of bacteria at genus level. **(d)** The relative abundance comparison of the six dominant bacterial genera with significant differences among the top 10. * represents significant difference (*p* < 0.05, ANOVA).

The ASVs were classified into 39 bacterial phyla and 1,019 genera. At the phylum level (Figure 1a), the relatively abundant groups included Proteobacteria, Acidobacteriota, Actinobacteriota, Gemmatimonadota, Bacteroidota, Firmicutes, Chloroflexi, and Myxococcota. Proteobacteria and Acidobacteriota were the dominant phyla, constituting 35.3 to 65.7% of the total bacteria, with a high abundance. Notably, Proteobacteria alone encompassed a significant proportion, from 19.4 to 52.8% of the total bacteria.

The analysis of phylum-level abundance variations among the three groups demonstrated significant differences (Figure 1b). Proteobacteria exhibited a significantly elevated abundance in wilt-affected soils (47.41%) compared to the unplanted and the non-wilt-affected soils (23.2 and 25.2%, respectively, *p* < 0.05, ANOVA). Acidobacteriota were more predominant in the control and non-wilt-affected soils (21.2 and 23.0%, respectively), with a substantial decrease in their abundance in the soils of wilted ginger plants (12.0%, *p* < 0.05, ANOVA). The abundance of Firmicutes did not show statistically significant differences across the three groups, potentially due to high variability within the data. Nonetheless, elevated abundance levels were noted in soils devoid of the small yellow ginger cultivation (12.8%), contrasting with a downward trend observed in the rhizosphere of both healthy and diseased ginger plants (1.0 and 0.9%, respectively). Additionally, the Chloroflexi phylum displayed a higher abundance specifically in the rhizosphere of healthy small yellow ginger plants (7.3%), significantly contrasting with the reduced abundance in both the unplanted and the wilt-affected soils (3.3 and 2.2%, respectively, *p* < 0.05, ANOVA).

At the genus level, the analysis highlighted several genera with considerable relative abundance, such as *Gemmatimonas*, *Candidatus_Solibacter*, *Ralstonia, Sphingomonas,* and Unclassified_Vicinamibacteriales (Figures 1c,d). In the rhizosphere of healthy small yellow ginger, *Gemmatimonas* and *Sphingomonas* displayed elevated relative abundances compared to the uncultivated soils (*p* < 0.05, ANOVA). Despite non-significant differences in their abundance between non-wilt-affected and wilt-affected soils, a quantitative reduction is apparent. *Candidatus_Solibacter* attained its maximum relative abundance in the soil surrounding healthy ginger (5.0%), with a subsequent abundance in unplanted soils (3.6%), and underwent a pronounced decline in diseased conditions (2.3%) relative to both unplanted and non-wilt-affected soils (*p* < 0.05, ANOVA). Unclassified_Vicinamibacteriales maintained relative abundances in both the uncultivated and the non-wilt-affected soils (3.4 and 2.3%, respectively); yet a significant diminution was observed in their soil prevalence after disease onset (1.0%). *Ralstonia*, a genus belonging to the Proteobacteria phylum, was detected in all three soil samples from diseased small yellow ginger, with relative abundances ranging from 2.0 to 26.7%. This is in stark contrast to its negligible relative abundance of less than 0.1% in the unplanted and the non-wilt-affected soils. Despite high variability in *Ralstonia* abundance within wilt-affected soils, which precluded statistical significance when compared to the non-wilt-affected soils, a pronounced quantitative increase in *Ralstonia* was observed in the wilt-affected soils, which was markedly higher than in both the uncultivated and the non-wilt-affected soils (Figure 1d).

### 3.3 Alpha-diversity analysis of soil microbial communities

**Figure 2:**
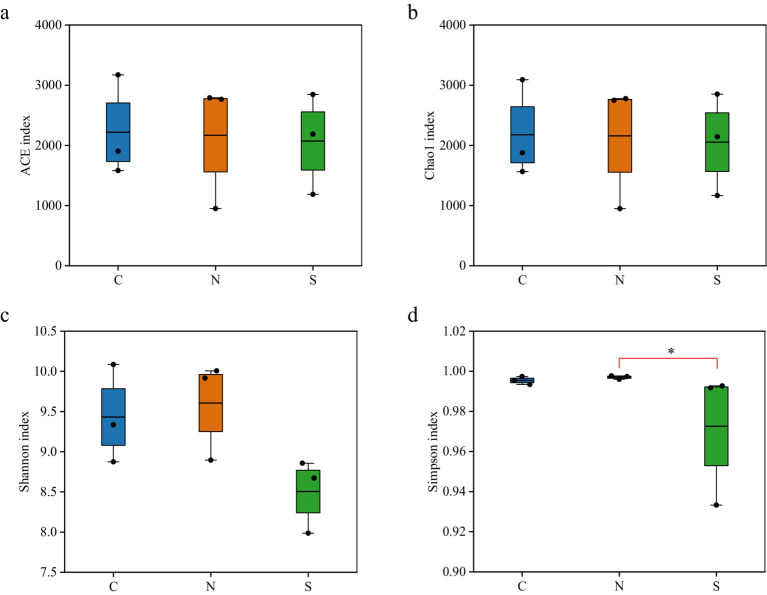
Boxplot of alpha-diversity indices. **(a)** Ace and **(b)** Chao1 indices reflect the abundance of ASVs in samples. **(c)** Shannon and **(d)** Simpson indices reflect the diversity of ASVs in samples. * represents significant difference (*p* < 0.05, KW test).

The diversity indices of the soil microbial communities revealed no significant disparities in the ACE values and Chao1 indices when comparing the wilt-affected ginger soil with the unplanted and non-wilt-affected soils, indicating similar levels of bacterial species richness among the soil samples (Figures 2a,b; Supplementary Table S1). However, the Shannon and Simpson diversity indices for the wilt-affected soil were observed to be lower in comparison to the unplanted and non-wilt-affected soils, with the Simpson index of the diseased ginger soil (0.9726) being significantly lower than that of the non-wilt-affected soil (0.9954, *p* < 0.05, KW test) (Figures 2c,d; Supplementary Table S1). These findings quantitatively demonstrated a reduced diversity within the rhizosphere soil of wilted ginger, reflecting a measurable impact on the bacterial communities present.

### 3.4 Beta-diversity and clustered abundance heatmaps

**Figure 3:**
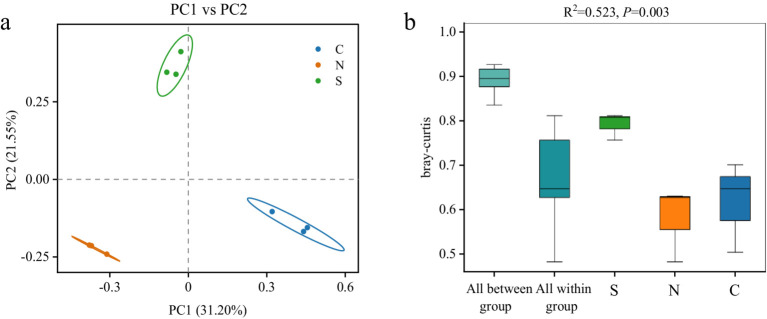
PCoA **(a)** and PERMANOVA **(b)** analysis of bacterial community structure based on the Bray–Curtis distance metric in all soil samples.

To assess differences in microbial community structure among groups, beta-diversity was analyzed based on Bray–Curtis dissimilarity (Gu et al., 2023). PCoA revealed clear separation between groups, with samples from the same condition clustering together and showing no inter-group overlap (Figure 3a), indicating distinct community structures. PERMANOVA further confirmed significant differences in community composition among groups (R^2^ = 0.523, *p* < 0.01, Figure 3b). The between-group dissimilarity substantially exceeded within-group variation, supporting the strong separation observed in ordination plots. Consistent with these findings, NMDS analysis yielded similar clustering results (Supplementary Figure S2). The stress value from the NMDS analysis was 0.0013, well below 0.05, indicating a high representativeness of the results (Dexter et al., 2018).

The beta-diversity results were further visually corroborated by a clustered abundance heatmap generated using row Z-score normalization of the top 100 bacterial genera (Euclidean distance, complete linkage; Supplementary Figure S3). Group S soils exhibited apparent heterogeneity in genus enrichment among replicates, whereas Group N soils demonstrated consistent enrichment patterns. Group C soils displayed intermediate variability. Despite intra-group variations, samples within each group maintained higher overall similarity in bacterial community composition compared to those between groups.

### 3.5 Ternary plot analysis

**Figure 4:**
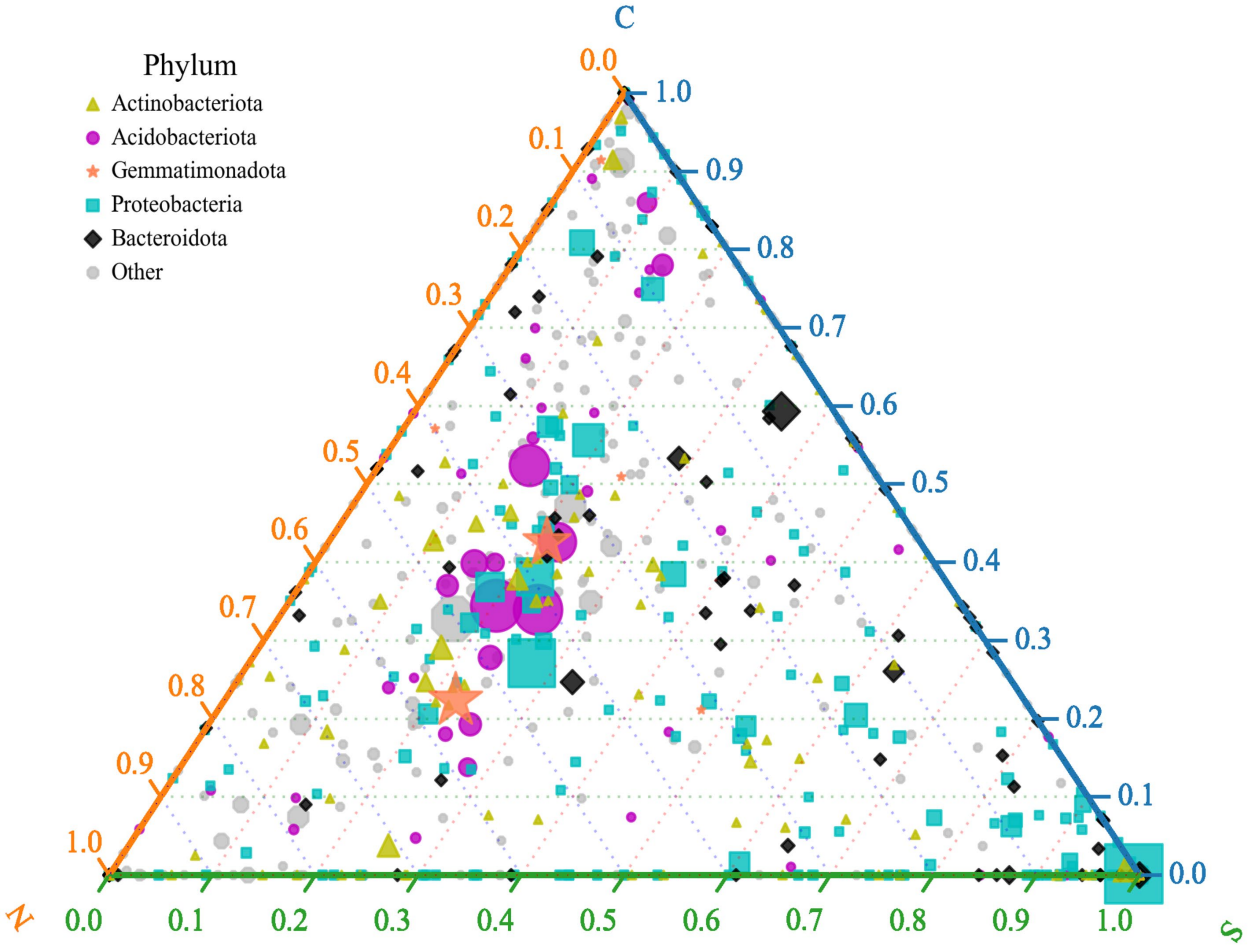
Ternary plot depicts different relative abundances of the genus across three sample groups (groups C, N, and S). Symbols represent individual genera, with size proportional to mean relative abundance across samples and color denoting phylum-level taxonomy.

The ternary plot illustrates the distribution of bacterial genera across three sample groups, focusing on the top five most abundant phyla (indicated by color in the legend; Figure 4). The position of a symbol indicates the relative proportional abundance of that genus within each group: proximity to a vertex signifies higher relative abundance in the corresponding group. The visualization reveals that the majority of bacterial genera within the phyla Acidobacteriota and Gemmatimonadota are markedly more abundant in rhizospheric soils from control (Group C) and healthy ginger plants (Group N) than in soils from wilted ginger plants (Group S). Genera classified within Proteobacteria and Bacteroidota are notably more abundant in Group S soils. In Group S soils, an increased abundance of certain bacterial genera within Proteobacteria is observed, particularly among genera positioned close to the Group S vertex. Notably, one genus exhibits particularly high abundance in this region, which is identified as the genus *Ralstonia* through inspection of the raw data from the ternary plot. This indicates that the significant increase in Proteobacteria abundance observed in Group S soils is predominantly driven by a substantial enrichment of the genus *Ralstonia* rather than by proportional increases in other Proteobacterial genera.

### 3.6 Comparative assessment of microbial biomarkers

**Figure 5:**
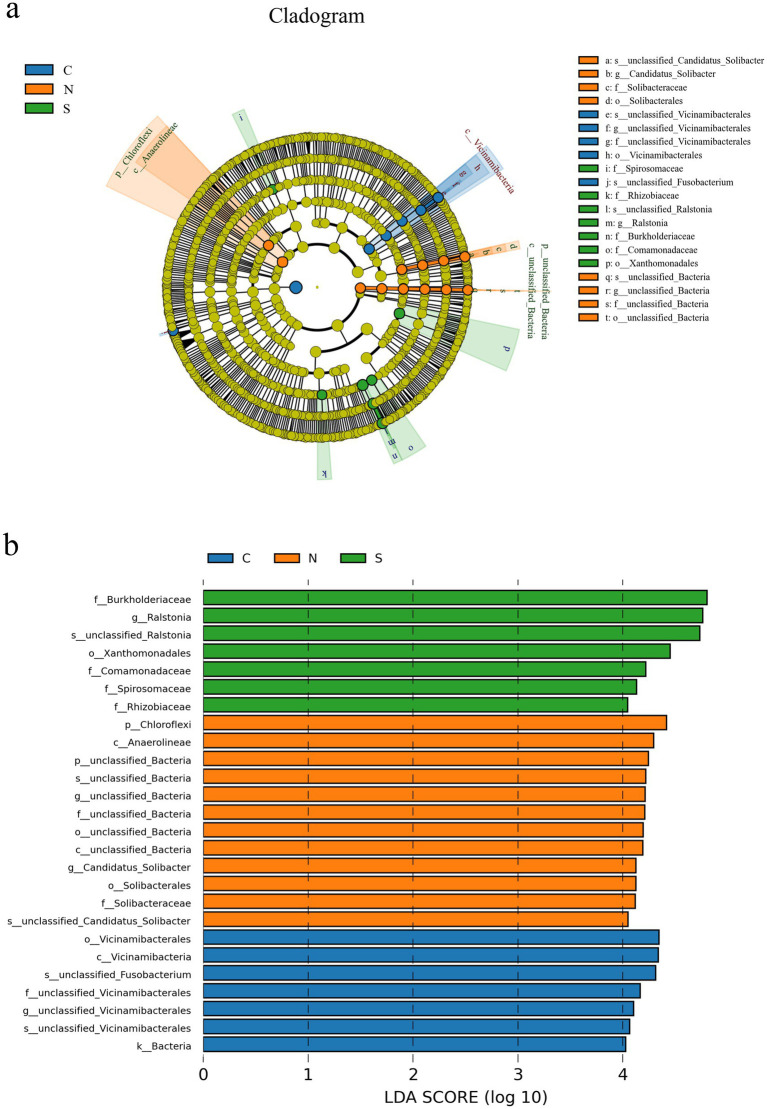
**(a)** LEfSe cladogram illustrating differentially abundant taxa across groups. **(b)** Bar plot of LDA effect sizes for significant microbial biomarkers (LDA score >4). **(a)** Circles from the center to outside layers represent taxonomic levels from phylum to species. Each node represents a specific taxon on the corresponding taxonomic level. The size of the dots is proportional to the relative abundance of that taxon in the group. Nodes colored yellow represent taxa that did not show statistically significant differential abundance among the groups. Otherwise, the nodes were colored according to the group with the highest relative abundance (LDA score >4). Different colors indicate different experimental groups; **(b)** Each bar represents a taxon identified as a biomarker with significant differential abundance and high discriminative power between groups. The length of the bar corresponds to the LDA score (effect size), indicating the magnitude of the difference in abundance. Different colors correspond to the group in which the taxon is most significantly enriched.

The LEfSe analysis, executed with an LDA score threshold of 4, revealed distinct biomarker taxa across the groups (Figure 5). Specifically, Figure 5a presents the cladogram (phylogenetic tree) generated by LEfSe, illustrating the taxonomic hierarchy of the features and highlighting those with significant differential abundance. The cladogram identified an unidentified species within the genus *Ralstonia* of the family Burkholderiaceae as a distinctive biomarker for Group S soils. Figure 5b depicts the LDA scores for the most discriminative features (potential biomarkers), quantifying the effect size of their abundance differences.

Beyond the primary *Ralstonia* (LDA = 4.69) biomarker for Group S soils, the LEfSe analysis also revealed that bacteria belonging to the families Comamonadaceae (LDA = 4.26), Spirosomaceae (LDA = 4.17), Rhizobiaceae (LDA = 4.06), and order Xanthomonadales (LDA = 4.49) exhibited higher relative abundance in Group S soils. Conversely, an unidentified *bacterium* (LDA = 4.39) within the order Vicinamibacterales and an unclassified *Fusobacterium* (LDA = 4.24) were more prevalent in Group C soils. In Group N soils, a higher relative abundance was observed for the class Anaerolineae (LDA = 4.30) of the phylum Chloroflexi, an unclassified genus *Candidatus Solibacter* (LDA = 4.17) of the family Soliacteraceae within the order Solibacterales, along with an unidentified bacterium from the domain *Bacteria*. Collectively, the LEfSe analysis reveals marked disparities in the biomarker taxa across the three sample groups.

### 3.7 Microbial community metabolic functions

**Figure 6:**
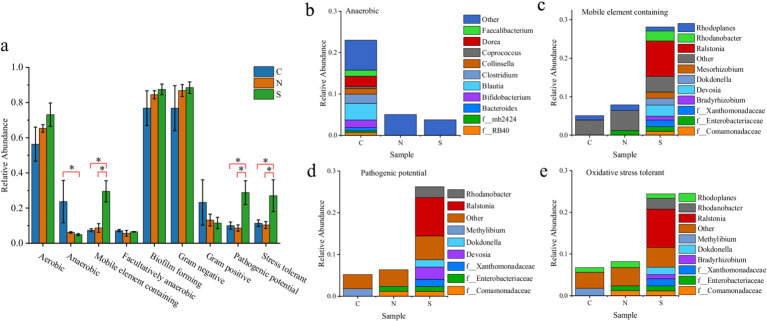
**(a)** Summary of BugBase-predicted bacterial phenotypes. * represents a significant difference. **(b–e)** Genus-level composition of bacteria associated with specific phenotypes per group: **(b)** aerobic, **(c)** mobile element-containing, **(d)** pathogenic potential, and **(e)** oxidative stress-tolerant.

Although a consistent increasing trend in aerobic abundance was noted from Group C to N to S, no statistically significant differences were observed among groups (Figure 6a). In contrast, anaerobic abundance was significantly reduced in the wilt-affected ginger soils (Group S) compared to the control soils (Group C) (*p* < 0.05, ANOVA; Figure 6a). While no significant difference was detected between the healthy ginger rhizosphere (Group N) and the control, pronounced shifts in anaerobic community composition occurred in both cultivated groups. Specifically, diverse anaerobic taxa prevalent in the uncultivated control soil (e.g., *Faecalibacterium*, *Coprococcus*, and *Blautia*) were largely absent in Groups N and S (Figure 6b). No significant differences in Gram-positive/Gram-negative ratios were detected among groups (*p* > 0.05, ANOVA). More importantly, BugBase predicted significantly higher relative abundance of mobile elements, oxidative stress tolerance, and pathogenic potential in Group S microbiota compared to other groups (*p* < 0.05, ANOVA; Figures 6c–e). A deeper investigation into the genera contributing substantially to these phenotypes identified *Ralstonia*, *Rhodanobacter*, *Dokdonella*, and *Devosia* as prominent. Notably, *Ralstonia* was the dominant taxon contributing to these predictions in Group S soils (Figures 6c–e).

## 4 Discussion

The structure and composition of soil microbial communities are intricately linked to the soil environment and are significantly influenced by crop cultivation and disease occurrence. In agricultural soils, bacteria and fungi are the predominant microbial groups. Our comprehensive analysis targeted both bacterial and fungal communities. Notably, despite the potential ability of certain fungi, such as the genus *Fusarium*, to induce wilt diseases (Lal et al., 2024), our data revealed no substantial increase in the genus *Fusarium* within wilt-affected soils (Supplementary Figure S9). Furthermore, no fungal genera exhibited significant proliferation, leading to the conclusion that fungi are unlikely to be the pathogens responsible for the ginger wilt disease in Rucheng County (Supplementary Figures S9, S13, and S14). Thus, our research focused on elucidating bacterial community dynamics.

In agricultural soils, the phyla Proteobacteria, Actinobacteriota, Bacteroidota, and Acidobacteriota constitute the predominant bacterial groups (Wang et al., 2023). Our study revealed that Proteobacteria and Acidobacteriota were the most abundant in the studied soils, with relative abundances ranging from 35.3 to 65.7%, aligning with previous findings (Yuan et al., 2021; Luo et al., 2023). Abundance analysis indicated that the cultivation of small yellow ginger and the incidence of wilt disease significantly altered the relative abundance of key bacterial phyla, specifically Proteobacteria, Acidobacteriota, Firmicutes, and Chloroflexi. Soils affected by wilt disease exhibited a notable increase in Proteobacteria and a corresponding decrease in Acidobacteriota, Firmicutes, and Chloroflexi. This shift aligns with a previous study on alterations in soil bacterial composition after ginger infection by *Ralstonia solanacearum* (Dang et al., 2023).

The observed changes in the soil microbial community suggest that both cultivation practices and disease occurrence exert substantial impacts on bacterial composition and overall community structure. Crucially, ternary plot analysis revealed that the significant increase in Proteobacteria abundance in wilt-affected soils was driven primarily by the enrichment of the genus *Ralstonia* (a known phytopathogen), rather than a broad increase across other Proteobacterial genera. This finding strongly indicates that the shift toward Proteobacteria dominance is specifically linked to the proliferation of this key pathogen. While we acknowledge the inherent functional diversity within Proteobacteria (including beneficial taxa such as *Pseudomonas fluorescens)* (Maheshwari and Sankar, 2023) and the existence of non-phytopathogenic *Ralstonia* strains in other contexts, the specific and dominant enrichment of *Ralstonia* observed here, coinciding with disease symptoms, points decisively to its pathogenic role. Conversely, the decrease in Acidobacteriota, Firmicutes, and Chloroflexi could signify a reduction in microbial functions associated with these groups, such as organic matter decomposition and nutrient cycling (Acidobacteriota) (Kalam et al., 2020), plant growth promotion and biocontrol (Firmicutes, notably *Bacillus* spp.) (Shen et al., 2023; Jabran et al., 2024), and participation in specific carbon cycling pathways (Chloroflexi) (Freches and Fradinho, 2024).

The relationship between wilted ginger plants and root bacterial diversity appears critical. Previous studies and our own diversity analysis consistently showed that ginger disease led to a decline in soil bacterial diversity (Liu et al., 2017; Wang et al., 2022a; Dang et al., 2023). Our data revealed that the biodiversity of wilt-affected soils was lower compared to both control soils and rhizosphere soils of healthy plants. This suggests that pathogens may disrupt the equilibrium of the rhizosphere microbiota. A less diverse community may be less capable of suppressing pathogen invasion or maintaining plant health, potentially creating conditions more favorable for the proliferation of *Ralstonia* and contributing to rhizosphere microecological imbalances (Dang et al., 2023). Further analyses (PCoA, NMDS, and heatmap) confirmed significant alterations in microbial community structure and composition in ginger-cultivated soils and wilt-affected soils. Notably, the rhizosphere microbiota of Rucheng’s small yellow ginger exhibits distinctive features when compared to previous studies on ginger wilt in other regions. This discrepancy suggests that the microbial consortia associated with ginger wilt may be strongly influenced by local edaphic conditions, agricultural practices, or host cultivar specificity. The distinct secondary dominant taxa observed here underscore that ginger wilt is not a monolithic disease but a context-dependent dysbiosis, highlighting the need for region-specific management strategies.

The potential mechanisms by which *Ralstonia* contributes to ginger wilt disease are well-documented (Jiang et al., 2017; Prameela and Suseela Bhai, 2020; Vailleau and Genin, 2023). *Ralstonia solanacearum* typically invades plant roots through wounds or natural openings, colonizes the xylem vessels, and rapidly multiplies. It produces exopolysaccharides (EPS) that occlude xylem vessels, blocking water transport and causing wilting. Additionally, it secretes cell wall-degrading enzymes and effector proteins that disrupt host cell function and suppress plant defenses. While the genus *Ralstonia* exhibits genetic diversity and includes non-pathogenic environmental strains, the dominant enrichment of *Ralstonia* in wilted ginger (supported by ternary plot and LEfSe analyses), coupled with its prominence in pathogenic phenotype predictions (Figure 6e), strongly suggests a pathogenic strain adapted to ginger. Further isolation and pathogenicity testing (e.g., Koch’s postulates) are warranted for confirmation. Beyond *Ralstonia*, LEfSe analysis (Figure 5) also identified other bacteria enriched in wilt-affected soils (Spirosomaceae, Rhizobiaceae, Comamonadaceae, and Xanthomonadales). While their roles in ginger wilt are less defined, they may contribute to disease dynamics; for example, some Comamonadaceae or Xanthomonadales members might thrive on decaying root material. Conversely, bacteria enriched in healthy plant soils (Anaerolineae and *Candidatus Solibacter*) may represent beneficial groups whose decline contributes to disease susceptibility.

Functional trait prediction via BugBase suggested that the diseased rhizosphere microbiome harbored a genomic potential indicative of a more pathogenic and resilient state. Crucially, the enhanced pathogenic potential, oxidative stress tolerance, and mobile element content were collectively driven by a network of co-enriched taxa. While *Ralstonia* was a key player, the genera *Rhodanobacter*, *Dokdonella*, and *Devosia* were also prominent contributors to these predicted traits. This collective enhancement suggests that these taxa are not merely bystanders but may actively facilitate the disease process. Thus, the severity of ginger wilt likely stems from the concerted action of a multigenus pathogenic alliance rather than from *Ralstonia* alone. It is important to note that these functional predictions are derived from 16S rRNA data and are constrained by limited sample size (*n* = 3 per group); they should therefore be interpreted as exploratory and hypothesis-generating. Despite these limitations, the predicted genomic traits provide a plausible mechanistic hypothesis for the severity and persistence of ginger wilt under field conditions. However, validation of *in situ* gene expression is required, warranting further investigation through metagenomic or metatranscriptomic approaches.

These findings have direct agricultural implications for managing ginger wilt in Rucheng County. First, pathogen-specific crop rotations should avoid planting ginger after known *Ralstonia solanacearum* hosts (e.g., Solanaceae crops like tomato, potato, pepper, and eggplant); instead, implementing extended rotations with non-host species, such as corn (maize), leafy vegetables, or legumes, is recommended to reduce pathogen load in the soil. Second, the observed reduction in beneficial phyla such as Acidobacteriota directly signals a degradation of soil health, impacting its capacity for organic matter decomposition. Therefore, soil health management strategies should prioritize practices that restore Acidobacteriota-mediated decomposition functions through organic amendments (compost, biochar), cover cropping, and reduced tillage to rebuild a suppressive microbiome. Third, the inferred functional synergy within the pathogenic consortium, including enhanced oxidative stress tolerance, suggests that biocontrol requires a multipronged approach. Introducing single antagonistic strains (e.g., *Bacillus* or *Pseudomonas*) may be insufficient. Instead, consortia of beneficial microbes should be developed to compete with, and disrupt the functionality of, the entire pathogenic network. Fourth, screening existing small yellow ginger landraces or developing new varieties with enhanced resistance to the local *Ralstonia* strain represents a sustainable solution. Finally, strict sanitation protocols must be implemented to prevent dissemination via contaminated tools, irrigation water, or soil movement.

## 5 Conclusion

This study demonstrates that ginger wilt in continuously cropped small yellow ginger significantly alters the composition and functional potential of soil microbial communities. We revealed a clear structural shift in the rhizospheric microbial community of diseased plants, which was significantly distinct from that of healthy plants. This dysbiosis was marked by a pronounced decline in beneficial phyla such as Acidobacteriota and Chloroflexi, concurrently with a significant increase in the abundance of the phylum Proteobacteria. Crucially, this expansion of Proteobacteria was primarily driven by the striking enrichment of a single genus, *Ralstonia,* which strongly implicates it as a key pathogen and diagnostic biomarker for ginger wilt disease. Beyond these taxonomic changes, functional prediction indicated that the diseased microbiome exhibits an enhanced genetic potential for pathogenicity and stress tolerance, and that this functional shift is contributed to not only by *Ralstonia* but also by other co-enriched taxa (e.g., *Rhodanobacter*, *Dokdonella*), suggesting disease severity may be mediated by a multigenus pathogenic alliance with *Ralstonia* as a keystone pathogen. These findings provide an ecological perspective for managing ginger wilt, emphasizing the need for strategies that rehabilitate overall microbiome function and resilience—through soil amendments and customized biocontrol—over those that target the pathogen alone. Future research should focus on isolating the ginger-adapted *Ralstonia* strain for pathogenicity validation and on empirically validating the predicted functional traits through molecular approaches.

## Data Availability

The datasets presented in this study can be found in online repositories. The names of the repository/repositories and accession number(s) can be found at: https://ngdc.cncb.ac.cn/bioproject/browse/PRJCA030785, PRJCA030785.
