# Supplementary material for: Wilt disease reshapes rhizosphere microbiota in small yellow ginger soils
Source: Front Microbiol. 2025 Oct 21;16:1670956. doi: 10.3389/fmicb.2025.1670956 (PMC12583060; doi:10.3389/fmicb.2025.1670956)
Supplement: Supplementary file 1 [file Data_Sheet_1.docx]

**Supplementary Material**

**PART A: Supplementary Materials for Bacterial Community Analysis**

**TABLE S1 Alpha-diversity indices of bacterial communities.**

| Sample ID | ACE | Chao1 | Simpson | Shannon |
| --- | --- | --- | --- | --- |
| C1 | 3171.424 | 3090.041 | 0.9974 | 10.0855 |
| C2 | 1903.166 | 1876.445 | 0.9954 | 9.3346 |
| C3 | 1581.311 | 1563.995 | 0.9934 | 8.8747 |
| N1 | 950.3093 | 949.0606 | 0.996 | 8.8958 |
| N2 | 2764.592 | 2776.796 | 0.9977 | 10.0067 |
| N3 | 2790.277 | 2749.364 | 0.9974 | 9.9161 |
| S1 | 1185.997 | 1167.063 | 0.9927 | 8.6716 |
| S2 | 2186.792 | 2143.689 | 0.9917 | 8.8578 |
| S3 | 2846.026 | 2850.684 | 0.9333 | 7.9866 |

FIGURE S1 Bacterial rarefaction curves based on 16S rRNA gene sequencing.

The x-axis represents the number of randomly sampled sequencing reads, and the y-axis denotes the corresponding number of ASVs obtained based on these reads. Each curve on the graph corresponds to an individual sample, marked with a distinct color for identification purposes.

FIGURE S2 NMDS analysis of bacterial community structure based on the Bray–Curtis distance metric in all soil samples.


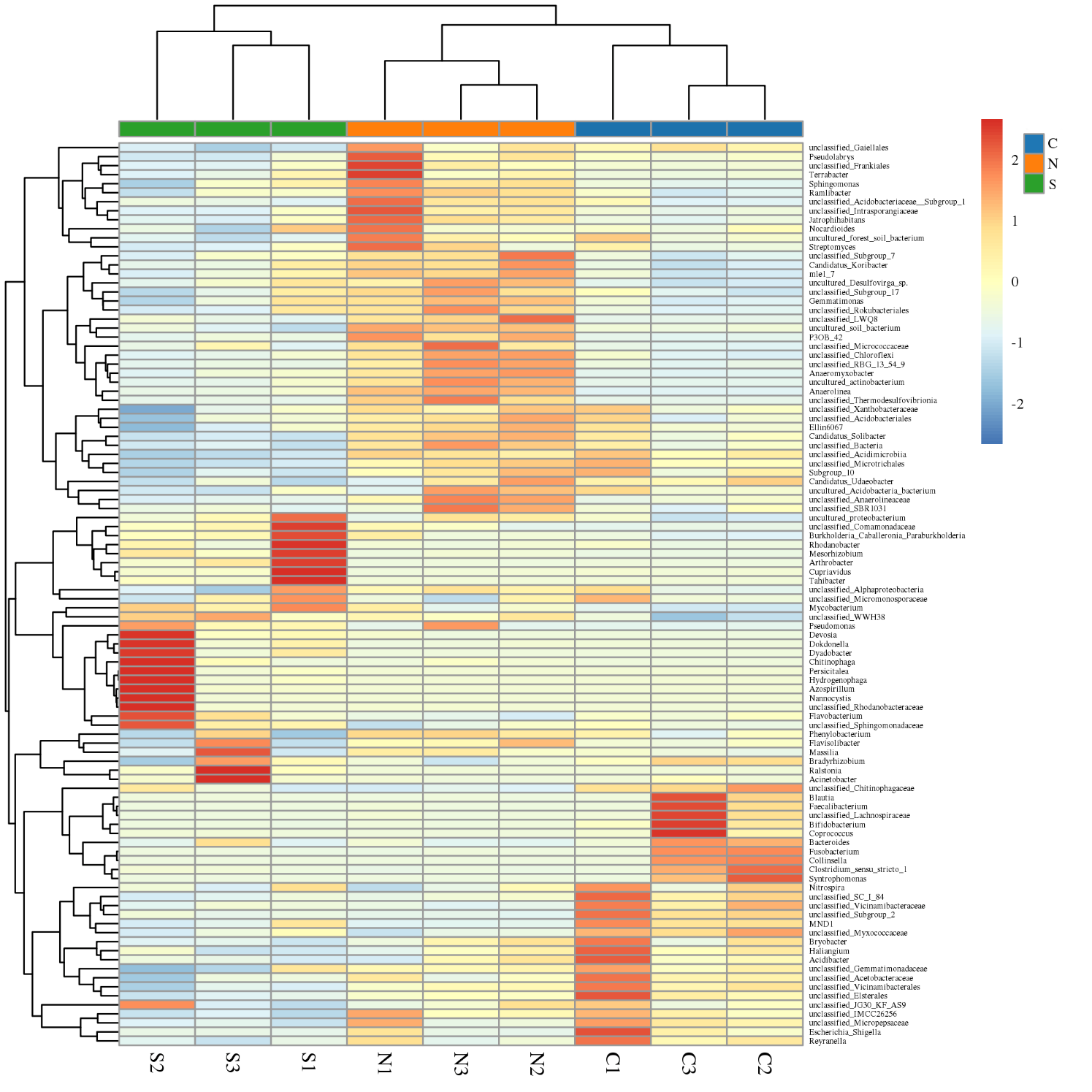


FIGURE S3 Clustered heatmap of genus-level enrichment patterns.

High Z-values indicate genera enriched in specific samples relative to other samples, not absolute abundance dominance. The sample clustering at the top of the diagram reflects similarity in taxonomic community composition among different samples(Xu et al., 2019).

**PART B: Supplementary Materials for Fungal Community Analysis**

**Materials and methods**

**Fungal ITS1 Gene Sequencing and Analysis**

To comprehensively characterize the microbial community and rule out fungal pathogens as a primary cause of ginger wilt, the ITS1 region of the fungal rRNA gene was also amplified and sequenced from the same DNA extracts used for bacterial 16S rRNA gene sequencing.

**ITS1 Amplification and Sequencing:**
The ITS1 region was amplified using the primer pair ITS1F/ITS2 (ITS1F: 5'-CTTGGTCATTTAGAGGAAGTAA-3'; ITS2: 5'-GCTGCGTTCTTCATCGATGC-3') (Yang et al., 2024). The amplification reaction system and thermal cycling conditions were identical to those used for bacterial 16S rRNA gene amplification, as described in the main text Methods. The resulting amplicons were purified, quantified, pooled, and sequenced on the Illumina NovaSeq 6000 platform alongside the 16S rRNA libraries.

**Fungal Data Processing:**
Raw ITS sequencing data were processed using a similar pipeline to the bacterial data. After quality filtering with Trimmomatic (v0.33) and primer removal with Cutadapt (v1.9.1), clean reads were denoised, merged, and chimera-checked using the DADA2 algorithm within QIIME2 to generate a table of Amplicon Sequence Variants (ASVs). Taxonomy was assigned to fungal ASVs using the Naive Bayes classifier in QIIME2 against the UNITE database (version 9.0) (Abarenkov et al., 2023) with a confidence threshold of 70%.

**Fungal Community Analysis:**
Standard alpha-diversity (e.g., Shannon index, Observed ASVs) and beta-diversity (e.g., PCoA based on Bray-Curtis dissimilarity) analyses were performed on the fungal ASV table using QIIME2. Differences in the relative abundance of major fungal phyla and genera (particularly known wilt pathogens such as *Fusarium*) between the Control (C), Non-wilt-affected (N), and Sick (S) groups were assessed using Kruskal-Wallis tests.

**TABLE S2 Summary of ITS sequencing data processing.**

| Sample ID | Raw Reads | Clean Reads | Denoised Reads | Merged Reads | Non-chimeric Reads |
| --- | --- | --- | --- | --- | --- |
| C1 | 55,659 | 55,161 | 53,361 | 50,630 | 49,200 |
| C2 | 62,292 | 61,763 | 59,684 | 56,645 | 55,215 |
| C3 | 54,507 | 54,181 | 51,598 | 48,954 | 47,426 |
| N1 | 64,139 | 63,955 | 61,949 | 59,958 | 59,137 |
| N2 | 65,023 | 64,790 | 62,753 | 60,406 | 59,344 |
| N3 | 66,724 | 66,513 | 64,912 | 63,280 | 62,054 |
| S1 | 50,470 | 50,080 | 48,567 | 46,332 | 45,298 |
| S2 | 59,760 | 59,297 | 57,533 | 55,785 | 54,742 |
| S3 | 52,348 | 52,185 | 50,664 | 49,056 | 48,478 |

**TABLE S3 Fungal alpha-diversity indices based on ITS sequencing.**

| Sample ID | ACE | Chao1 | Simpson | Shannon |
| --- | --- | --- | --- | --- |
| C1 | 399.8746 | 399.1667 | 0.9607 | 6.4544 |
| C2 | 538.5604 | 538.2353 | 0.9717 | 6.7632 |
| C3 | 635.2212 | 635 | 0.983 | 7.2947 |
| N1 | 568.9462 | 567.275 | 0.9513 | 6.0168 |
| N2 | 572.5094 | 573.7727 | 0.9234 | 5.6737 |
| N3 | 412.0853 | 410.7742 | 0.9102 | 4.9064 |
| S1 | 372 | 372 | 0.9522 | 5.8963 |
| S2 | 502.3379 | 502.2353 | 0.9765 | 7.0464 |
| S3 | 476.8655 | 476.2222 | 0.9109 | 5.1988 |

FIGURE S4 Fungal rarefaction curves based on ITS region sequencing.

FIGURE S5 Boxplot of fungal Alpha-diversity indices. (a) Ace and (b) Chao1 indices reflect the ASVs abundance in samples. (c) Shannon and (d) Simpson indices reflect the diversity of ASVs in samples.

FIGURE S6 The fungal community composition at the phylum level.

FIGURE S7 The relative abundance comparison of the top 10 phyla among different samples.

FIGURE S8 Main community composition of fungal at genus level.

FIGURE S9 The relative abundance comparison of the top 10 fungal genera. * represents significant difference (*P* < 0.05, ANOVA).


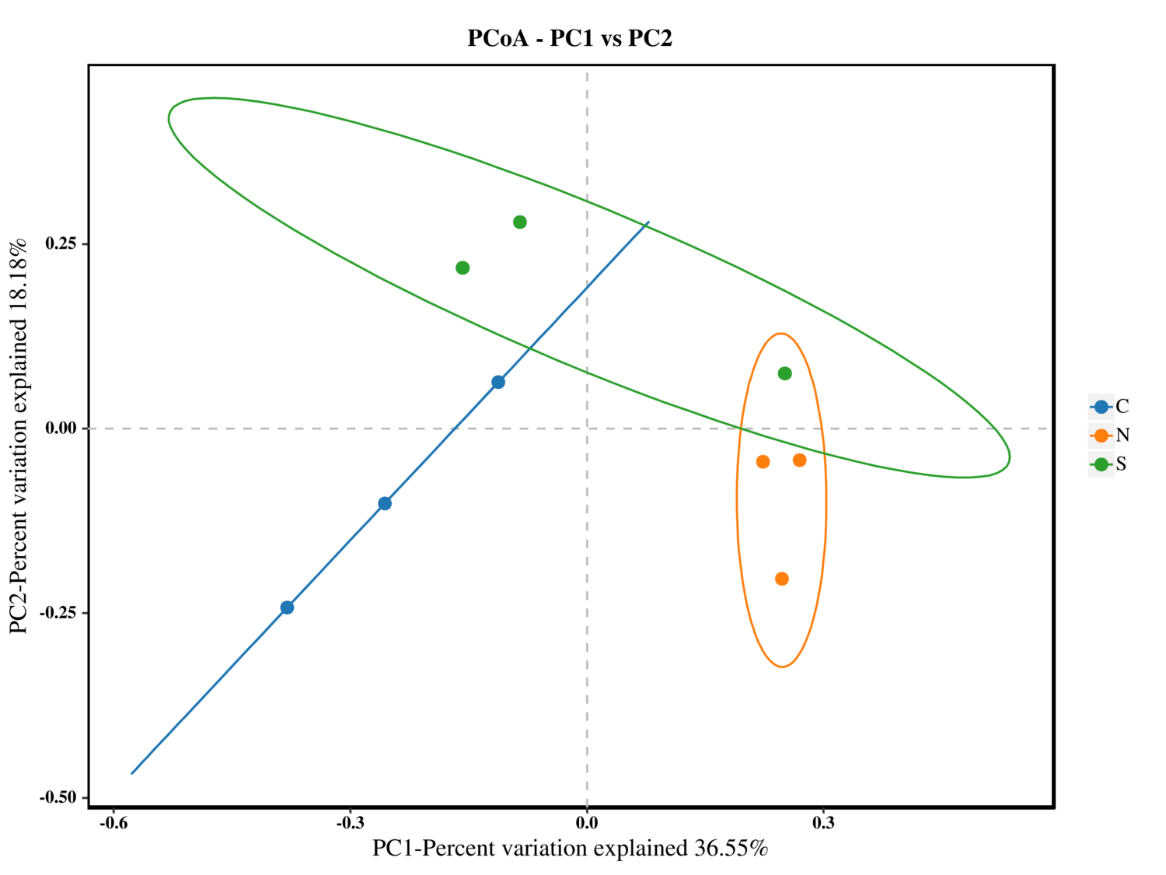


FIGURE S10 PCoA analysis of fungal community structure based on the Bray–Curtis distance metric in all soil samples.


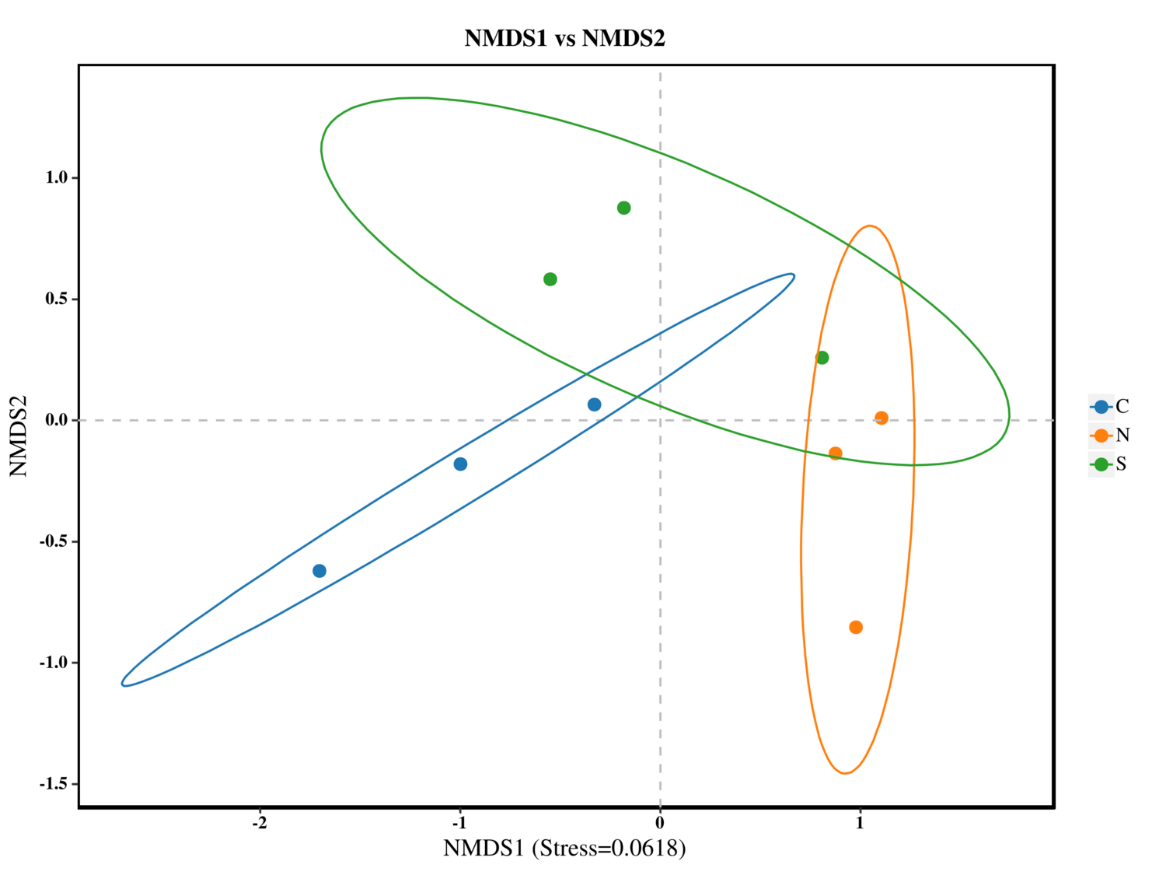


FIGURE S11 NMDS analysis of fungal community structure based on the Bray–Curtis distance metric in all soil samples.


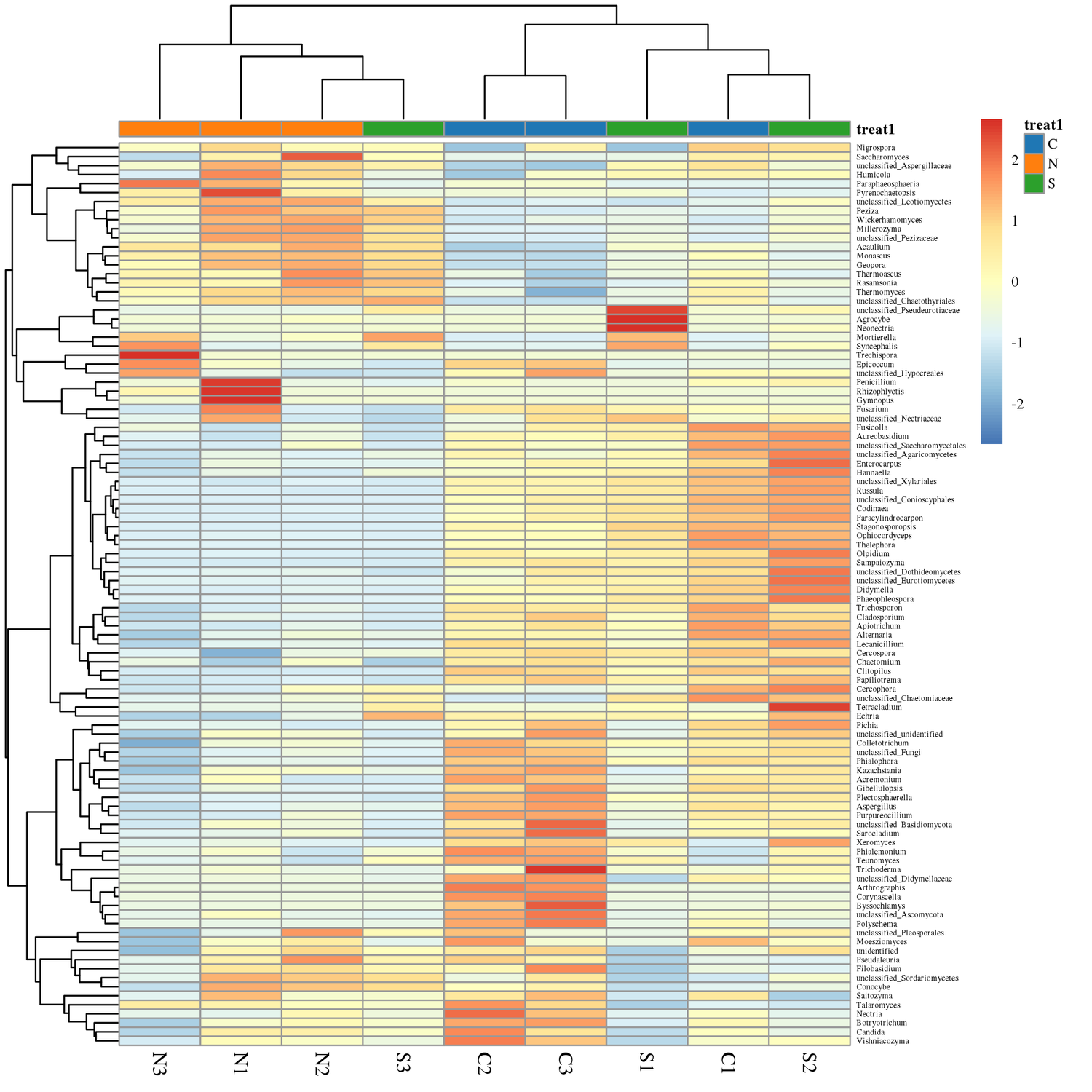


FIGURE S12 Clustered heatmap of genus-level enrichment patterns.


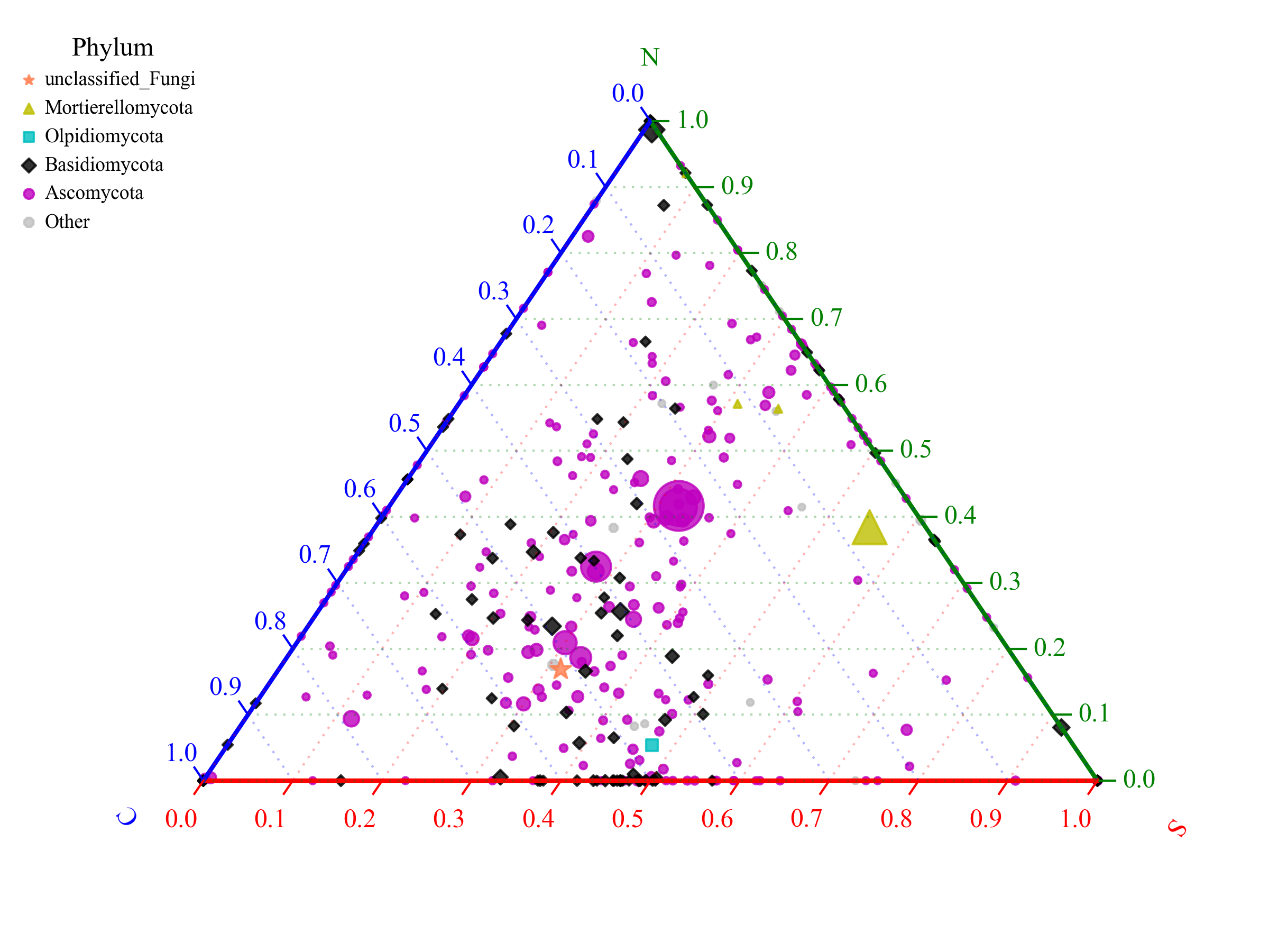


FIGURE S13 Ternary plot depicts different relative abundances of the genus across three sample groups (Group C, N, S). Symbols represent individual genera, with size proportional to mean relative abundance across samples and color denoting phylum-level taxonomy.


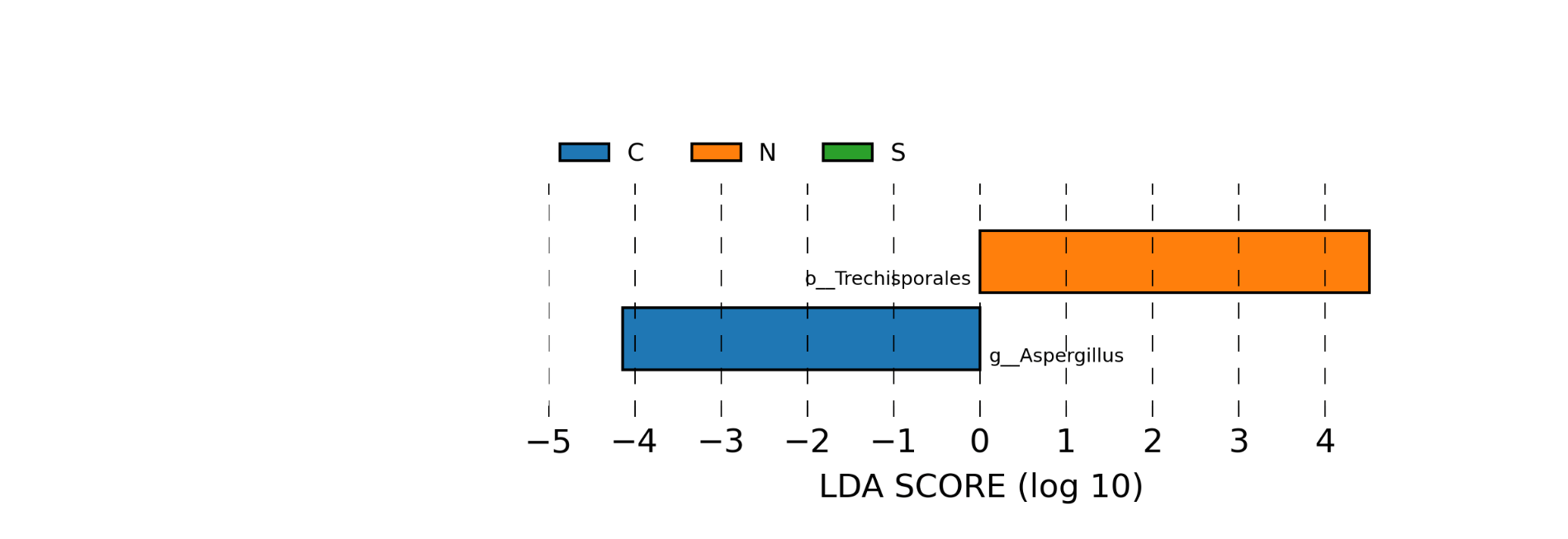


FIGURE S14 Bar plot of LDA effect sizes for significant microbial biomarkers (LDA score >4 ).

**References**

Abarenkov, K., Nilsson, R. H., Larsson, K.-H., Taylor, A. F. S., May, T. W., Frøslev, T. G., et al. (2024). The UNITE database for molecular identification and taxonomic communication of fungi and other eukaryotes: sequences, taxa and classifications reconsidered. Nucleic Acids Res. 52, D791–D797. doi: 10.1093/nar/gkad1039

Xu, G., Xing, W., Li, T., Xue, M., Ma, Z., Jiang, N., et al. (2019). Comparative study on the effects of different feeding habits and diets on intestinal microbiota in Acipenser baeri Brandt and Huso huso. BMC Microbiol. 19, 297. doi: 10.1186/s12866-019-1673-6

Yang, X., Xiong, J., Du, T., Ju, X., Gan, Y., Li, S., et al. (2024). Diversifying crop rotation increases food production, reduces net greenhouse gas emissions and improves soil health. Nat. Commun. 15, 198. doi: 10.1038/s41467-023-44464-9
